# Mucosal immunization with PspA (Pneumococcal surface protein A)-adsorbed nanoparticles targeting the lungs for protection against pneumococcal infection

**DOI:** 10.1371/journal.pone.0191692

**Published:** 2018-01-23

**Authors:** Tasson C. Rodrigues, Maria Leonor S. Oliveira, Alessandra Soares-Schanoski, Stefanni L. Chavez-Rico, Douglas B. Figueiredo, Viviane M. Gonçalves, Daniela M. Ferreira, Nitesh K. Kunda, Imran Y. Saleem, Eliane N. Miyaji

**Affiliations:** 1 Laboratório de Bacteriologia, Instituto Butantan, São Paulo, SP, Brazil; 2 Laboratório Especial de Desenvolvimento de Vacinas, Instituto Butantan, São Paulo, SP, Brazil; 3 Respiratory Infection Group, Liverpool School of Tropical Medicine, Liverpool, United Kingdom; 4 Formulation and Drug Delivery Research, School of Pharmacy and Biomolecular Sciences, Liverpool John Moores University, Liverpool, United Kingdom; Universidad Nacional de la Plata, ARGENTINA

## Abstract

Burden of pneumonia caused by *Streptococcus pneumoniae* remains high despite the availability of conjugate vaccines. Mucosal immunization targeting the lungs is an attractive alternative for the induction of local immune responses to improve protection against pneumonia. Our group had previously described the development of poly(glycerol adipate-co-ω-pentadecalactone) (PGA-co-PDL) polymeric nanoparticles (NPs) adsorbed with Pneumococcal surface protein A from clade 4 (PspA4Pro) within L-leucine microcarriers (nanocomposite microparticles—NCMPs) for mucosal delivery targeting the lungs (NP/NCMP PspA4Pro). NP/NCMP PspA4Pro was now used for immunization of mice. Inoculation of this formulation induced anti-PspA4Pro IgG antibodies in serum and lungs. Analysis of binding of serum IgG to intact bacteria showed efficient binding to bacteria expressing PspA from clades 3, 4 and 5 (family 2), but no binding to bacteria expressing PspA from clades 1 and 2 (family 1) was observed. Both mucosal immunization with NP/NCMP PspA4Pro and subcutaneous injection of the protein elicited partial protection against intranasal lethal pneumococcal challenge with a serotype 3 strain expressing PspA from clade 5 (PspA5). Although similar survival levels were observed for mucosal immunization with NP/NCMP PspA4Pro and subcutaneous immunization with purified protein, NP/NCMP PspA4Pro induced earlier control of the infection. Conversely, neither immunization with NP/NCMP PspA4Pro nor subcutaneous immunization with purified protein reduced bacterial burden in the lungs after challenge with a serotype 19F strain expressing PspA from clade 1 (PspA1). Mucosal immunization with NP/NCMP PspA4Pro targeting the lungs is thus able to induce local and systemic antibodies, conferring protection only against a strain expressing PspA from the homologous family 2.

## Introduction

Despite the availability of vaccines, *Streptococcus pneumoniae* remains an important cause of community acquired pneumonia and invasive pneumococcal disease (IPD). Polysaccharide conjugated vaccines confer good levels of protection against IPD caused by vaccine serotypes, but studies evaluating efficacy against pneumonia show varying results [1, 2]. It has been estimated that 2.7 million deaths occurred globally in 2015 due to lower respiratory infections, of which 704,000 occurred among children younger than 5 years. Pneumococcal pneumonia accounted for 55.8% of deaths due to lower respiratory infection in children younger than 5 years [3], which clearly shows that new vaccine strategies to control the burden of pneumococcal pneumonia are needed. The use of vaccine formulations that induce local immune responses in the lungs could be an interesting approach. Interest in lung vaccination has recently increased for prevention and treatment of respiratory diseases such as tuberculosis and influenza [4–6]. Besides the induction of local immune responses, vaccines targeting the lungs would have other advantages over parenteral injection, such as no risks of needle injuries and removal of cold chain requirements in the case of dry powder formulations [7].

Protein antigens can be an interesting alternative for pneumococcal polysaccharide vaccines and a promising candidate is Pneumococcal surface protein A (PspA). PspA is an important virulence factor and parenteral immunization with this antigen has been shown to be protective in pneumococcal challenge models of lethal infection and pneumonia [8–10]. PspA shows variability in different isolates and sequence-based classification divides PspA variants into three families, that are further subdivided into six clades: family 1 (clades 1 and 2), family 2 (clades 3, 4 and 5) and family 3 (clade 6) [11]. To achieve complete coverage, it has been suggested that a PspA-based vaccine should contain at least one PspA from each of the two major families (families 1 and 2) [11]. Our group has previously shown that parenteral immunization of mice with a recombinant PspA from clade 4 (PspA4, family 2) or from clade 5 (PspA5, family 2) using alum as adjuvant induces protection against lethal pneumococcal challenge with strains expressing PspA from families 1 and 2 [12]. Intranasal vaccination with PspA against pneumococcal infection has also been tested in animal models. Intranasal immunization of mice with native PspA using cholera toxin B-subunit or a non-toxic cholera toxin mutant as adjuvant was shown to elicit humoral responses in serum and saliva and protection against lethal challenge with a serotype 3 strain [13, 14]. More recently, a nanogel containing recombinant PspA was shown to protect mice against a lethal pneumococcal challenge after intranasal immunization of mice [15]. Nasal delivery of an optimized nanogel containing recombinant PspA to rhesus macaques was later shown to induce IgG in serum and bronchoalveolar lavage fluid (BALF), IgA in nasal wash and a Th2/Th17 cytokine response [16].

Our group has recently described the development of a dry powder formulation containing recombinant PspA from clade 4 (PspA4Pro) adsorbed onto the surface of poly(glycerol adipate-co-ω-pentadecalactone) (PGA-co-PDL) polymeric nanoparticles (NPs) encapsulated in L-leucine microparticles. The aim was to use these dry powder nanocomposite microparticles (NCMPs) as a pulmonary vaccine that can be delivered to the lungs via an inhaler or nebulizer to humans. The NP/NCMPs containing PspA4Pro (NP/NCMP PspA4Pro) were shown to be ~2 μm particles and *in vitro* lung deposition data suggested delivery to the bronchial-alveolar region of the lungs. Furthermore, *in vitro* internalization by DCs confirmed suitability of the size of the produced NPs (~300 nm) for vaccine delivery [17]. Nano- and microparticles containing recombinant PspA have been previously tested in mice using parenteral immunization. Spray-dried polylactide (PLA) microparticles entrapping recombinant PspA were tested as intramuscular immunization admixed with alum, eliciting a robust IgG response in mice [18]. Polyanhydride nanoparticles were also used to encapsulate recombinant PspA and subcutaneous immunization with these particles elicited an antibody response with high titer and avidity [19]. This work now describes the immune responses and protection potential of mucosal immunization with NP/NCMP PspA4Pro based on PGA-co-PDL targeting the lungs of mice. To our knowledge, this is the first study targeting the lungs for immunization with nanoparticles containing a protein antigen against pneumococcal infection.

## Materials and methods

### Ethics statement

This study was performed according to the guidelines outlined by the Brazilian National Council for Control of Animal Experimentation (CONCEA). Experimental protocols were approved by the Ethic Committee on Animal Use of the Butantan Institute (CEUAIB) under protocol number 1160/13. Five to six animals were housed per cage inside a ventilated cabinet under controlled temperature and light cycle (12/12 hours, light/dark cycle) with daily monitoring in a BSL2 animal facility. Food and water were given ad libitum. Monitoring and manipulation was done by trained personnel.

### Bacterial strains and growth conditions

*S*. *pneumoniae* strains were grown in Todd-Hewitt broth (Difco) supplemented with 0.5% yeast extract (THY) at 37°C without shaking. Bacteria were always plated in blood agar and grown overnight at 37°C before culture in THY. Stocks were maintained at −80°C in THY containing 20% glycerol.

### Preparation of NP/NCMPs adsorbed with PspA4Pro

The expression and purification of PspA4Pro (PspA clade 4, encompassing mature N-terminal region till proline-rich region) was described previously [20]. Briefly, *Escherichia coli* BL21 DE3 transformed with pET-pspA4Pro was used to express the protein. Purification was performed through double ion exchange chromatography. With this method, PspA4Pro was recovered with a high purity (greater than 97%) and low endotoxin concentration (0.07 EU/μg PspA4Pro). PGA-co-PDL NPs were adsorbed with PspA4Pro and formulated as NCMPs with L-leucine through spray-drying as previously described [17].

### Immunization of mice with NP/NCMPs

Five- to seven-week-old female specific-pathogen-free (SPF) BALB/c mice (5–6 animals per group) were obtained from the Medical School of the University of São Paulo (São Paulo, Brazil). Mucosal immunization targeting the lungs was conducted after anesthesia of mice by intraperitoneal (ip) inoculation of a xylazine/ketamine solution (20 mg/Kg of xylazine and 100 mg/Kg of ketamine). For delivery of NP/NCMPs (NP/NCMP empty or NP/NCMP PspA4Pro—containing 2 μg PspA4Pro), formulations were resuspended in saline (50 μl/dose) immediately before instillation into one nostril using a micropipette. Nasal instillation of this large volume under anesthesia ensures delivery of the majority of the inoculum to the lungs of mice (S1 Fig). Animals injected with saline or purified PspA4Pro subcutaneously (sc) (5 μg in 100 μl) and with purified PspA4Pro instilled into the lungs (5 μg in 50 μl, instillation into one nostril under anesthesia) were used as controls. Mice were immunized twice with a 14-days interval. Duration of experiments was 28 to 45 days, depending on whether animals were challenged or not.

### Collection of serum and BALF

Serum was collected 14 days after each dose for the evaluation of IgG levels. For collection of BALF, mice were euthanized through the ip route with a lethal dose of a xylazine/ketamine solution (60 mg/Kg of xylazine and 300 mg/Kg of ketamine). BALF was collected, as previously described [21], for the evaluation of IgG, IgA, cytokine/chemokine content and immunophenotyping of cells.

### Measurement of antibodies by enzyme-linked immunosorbent assay (ELISA)

ELISA was carried out as described previously [12] in plates coated with 5 μg/ml PspA4Pro. For the detection of serum antibodies, goat anti-mouse IgG conjugated with horseradish peroxidase (Sigma-Aldrich) was used as secondary antibody. For IgG isotyping, goat anti-mouse IgG1, goat-anti-mouse IgG2a and rabbit anti-goat IgG conjugated with horseradish peroxidase (Southern Biotech) were used. The titer was defined as the reciprocal of the highest dilution with an *A*_492_ ≥ 0.1. For the detection of antibodies in BALF, goat anti-mouse IgG conjugated with alkaline phosphatase (AP), goat anti-mouse IgA and rabbit anti-goat IgG conjugated with AP (Southern Biotech) were used and *A*_*405*_ was measured. Background value of each plate (0.040 to 0.045) was subtracted from the actual absorbance obtained for each sample.

### Binding of antibodies to intact bacteria

Antibody binding was performed as previously described [22]. Strains EF3030 (serotype 19F, PspA1), D39 (serotype 2, PspA2), M10 (serotype 11A, PspA3), 3JYP2670 (serotype 3, PspA4) and ATCC6303 (serotype 3, PspA5) [9, 12] were plated on blood agar for overnight growth, then cultured in THY to OD_600 nm_ 0.4–0.5 (~10^8^ CFU/ml) and harvested by centrifugation. Bacteria were washed, suspended in PBS and incubated with 1% of pooled sera for 30 min at 37°C. Samples were washed once with PBS before incubation with fluorescein isothiocyanate (FITC)-conjugated anti-mouse IgG (Sigma) for 30 min on ice. Samples were fixed with 2% formaldehyde after two washing steps and stored at 4°C. Flow cytometry analysis was conducted using FACSCanto (BD Biosciences), and 10,000 gated events were recorded.

### Pneumococcal challenge

Twenty one days after the last immunization, mice were anesthetized through the ip route with a xylazine/ketamine solution (20 mg/Kg of xylazine and 100 mg/Kg of ketamine) and challenged with 5x10^4^ CFU of strain ATCC6303 or 10^7^ CFU of strain EF3030. A volume of 50 μl was inoculated into one nostril. Mice were euthanized 12 or 24 hours later to collect BALF. Pneumococcal load was determined by plating serial dilutions of the samples on blood agar with 4 μg/ml gentamicin. For the analysis of overall survival, mice were evaluated for 10 days after challenge. Animals were monitored twice daily after challenge and lethargic animals with reduced ability to move were euthanized immediately through the ip route with a lethal dose of a xylazine/ketamine solution (60 mg/Kg of xylazine and 300 mg/Kg of ketamine). No animals died before meeting criteria for euthanasia.

### Measurement of cytokine/chemokine in BALF

A Luminex-based assay (Milliplex Map Mouse Cytokine/Chemokine Magnetic Bead Panel, Merck Millipore) was used to measure IFN-γ, IL-1-β, IL-5, IL-6, IL-10, IL-17A, KC/CXCL1, MCP-1/CCL2, MIP-2/CXCL2 and TNF-α in BALF samples. Results were acquired using Magpix (Merck Millipore) with Xponent (Luminex software) and analyzed with Milliplex Analyst (Merck Millipore), based on standard curves plotted through a 5-parameter logistic curve setting.

### Immunophenotyping of BALF cells

Cells recovered from BALF samples were immunophenotyped using anti-F4/80 PE-Cy7, anti-CD11c APC, anti-CD11b BB515, anti-CD4 APC-Cy7, anti-CD8 PerCP-Cy5.5, anti-B220 PE, anti-Ly6G BV421 (BD Biosciences) using FACSCanto II (BD Biosciences). The cytometer was set up using CompBeads (BD Biosciences) according to standard procedures. 30,000 events were recorded, excluding the low complexity events, through the FSC and SSC parameters, and dead cells by using the FVS-510 marker (BD Biosciences). The analysis was performed using FlowJo V10 software.

### Statistical analysis

Statistical analysis was performed using Prism 5.02 (GraphPad). Differences between groups were analyzed using One-way Analysis of Variance (ANOVA) with Tukey’s Multicomparison Test, Unpaired t-test or Paired t-test. Analysis of survival was performed using Fisher Exact Test and Log-rank (Mantel-Cox) test. * and #—*p*≤0.05, ** and ##—*p*≤0.01, ***—*p*≤0.001.

## Results

### Induction of anti-PspA4Pro antibodies by immunization with NP/NCMPs

**Fig 1:**
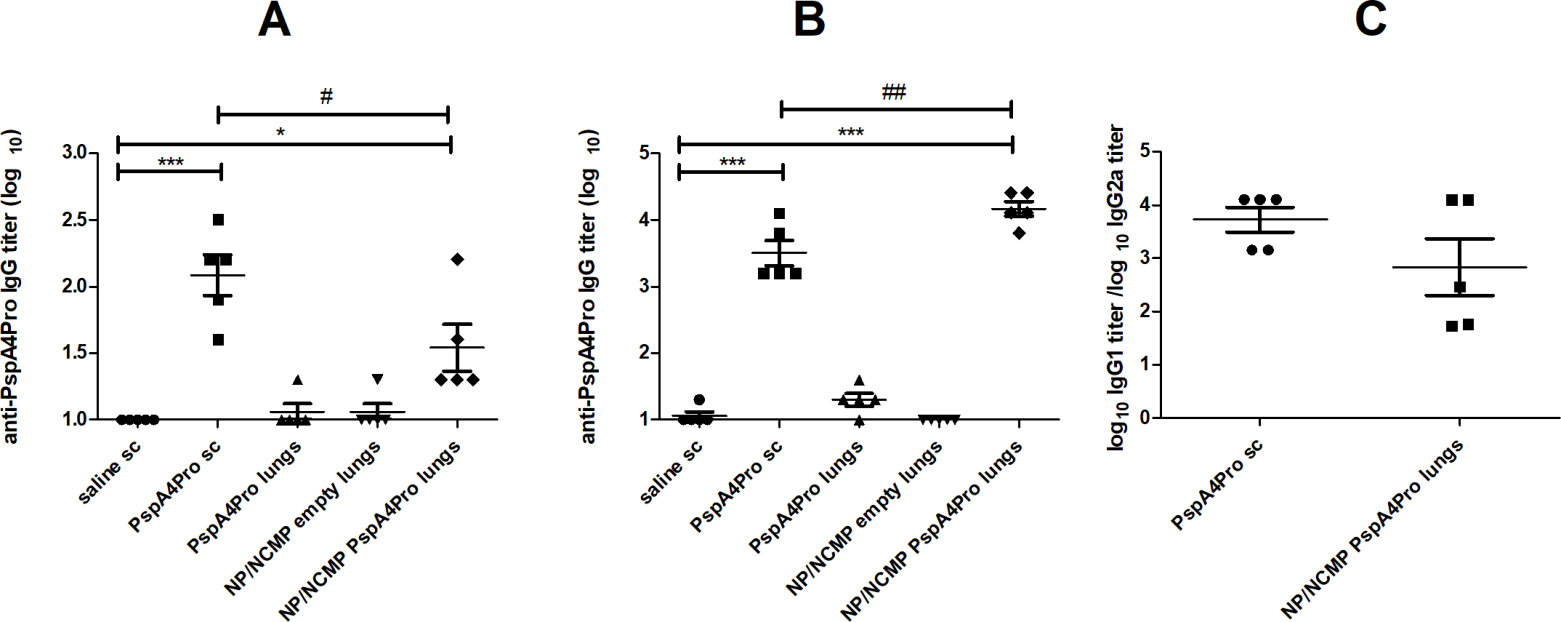
Induction of serum anti-PspA4Pro IgG antibodies by mucosal immunization targeting the lungs. The induction of anti-PspA4Pro IgG antibodies in sera from mice inoculated with the indicated formulations was determined by ELISA. Mice were inoculated with 1 (A) or 2 (B and C) doses of the formulations. Control mice were inoculated sc with saline or PspA4Pro. Log_10_ anti-PspA4Pro IgG titers (A and B) and log_10_ anti-PspA4Pro IgG1 titer/log_10_ anti-PspA4Pro IgG2a titer ratios (C) are shown. * indicates statistically significant difference with saline and # indicates statistically significant difference with PspA4Pro sc (One-way ANOVA, Tukey’s Multicomparison Test for A and B; Unpaired t-test for C). Symbols represent each individual. Means±standard errors are shown. Representative of at least two independent experiments.

**Fig 2:**
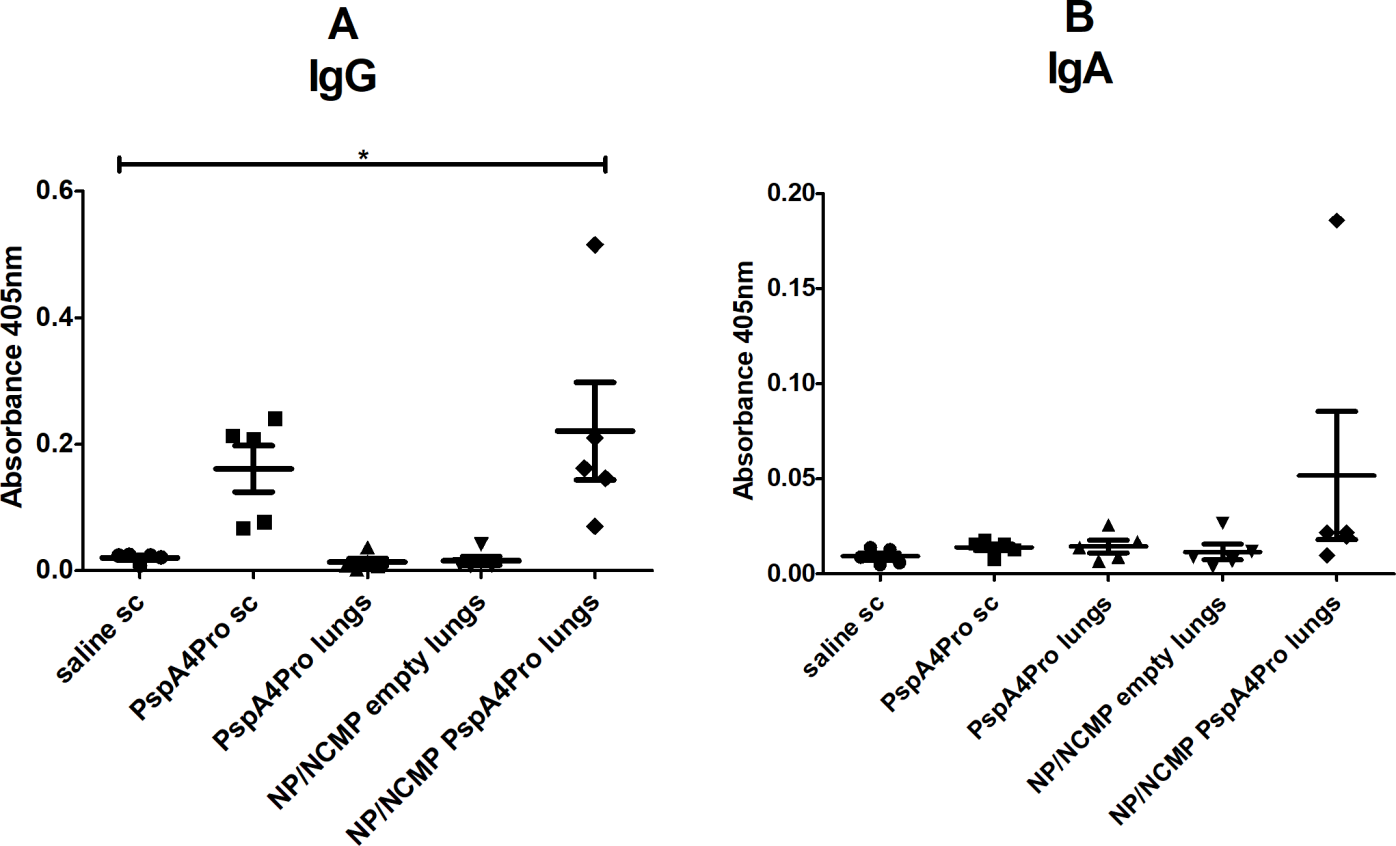
Induction of anti-PspA4Pro antibodies in BALF by mucosal immunization targeting the lungs. The induction of anti-PspA4Pro IgG (A) and IgA (B) antibodies in BALF from mice inoculated with the indicated formulations was determined by ELISA. Mice were inoculated with 2 doses of the formulations. Control mice were inoculated sc with saline or PspA4Pro. *A*_*405*_ nm of samples diluted 1:2 is shown. * indicates statistically significant difference with saline (One-way ANOVA, Tukey’s Multicomparison Test). Symbols represent each individual. Means±standard errors are shown.

Mucosal immunization targeting the lungs was done with the resuspension of NP/NCMP PspA4Pro. Purified protein was used as control in parenteral (PspA4Pro sc) and mucosal (PspA4Pro lungs) immunizations. Mice received two doses of the formulations with a 15-day interval and anti-PspA4Pro IgG titers were measured in the serum obtained from blood samples collected 14 days after each dose. We observed a significant increase of IgG titers in serum of mice immunized with PspA4Pro sc and NP/NCMP PspA4Pro compared with saline after the first (Fig 1A) and second doses (Fig 1B). Negligible antibody response was observed after mucosal immunization with the purified protein in the group PspA4Pro lungs. Immunization with NP/NCMP PspA4Pro elicited lower responses than PspA4Pro sc after the first dose, but a robust antibody response was observed after the second dose, with titers higher than those elicited by PspA4Pro sc (Fig 1B). Both PspA4Pro sc and NP/NCMP PspA4Pro elicited significantly higher titers of anti-PspA4Pro IgG1 compared with IgG2a (*p*≤0.01; Paired t-test), indicating a bias toward a Th2 response. No differences in IgG1/IgG2a ratios were observed between animals immunized with PspA4Pro sc and NP/NCMP PspA4Pro (Fig 1C). Twenty one days after the second dose, BALF was collected and anti-PspA4Pro IgG and IgA were measured. NP/NCMP PspA4Pro showed significantly higher levels of IgG in BALF than saline. Moreover, PspA4Pro sc induced a slight but not significant increase in IgG titers (Fig 2A), whereas IgA levels were not increased in any of the groups (Fig 2B).

### Binding of anti-PspA4Pro IgG to pneumococcal strains expressing different PspAs

**Fig 3:**
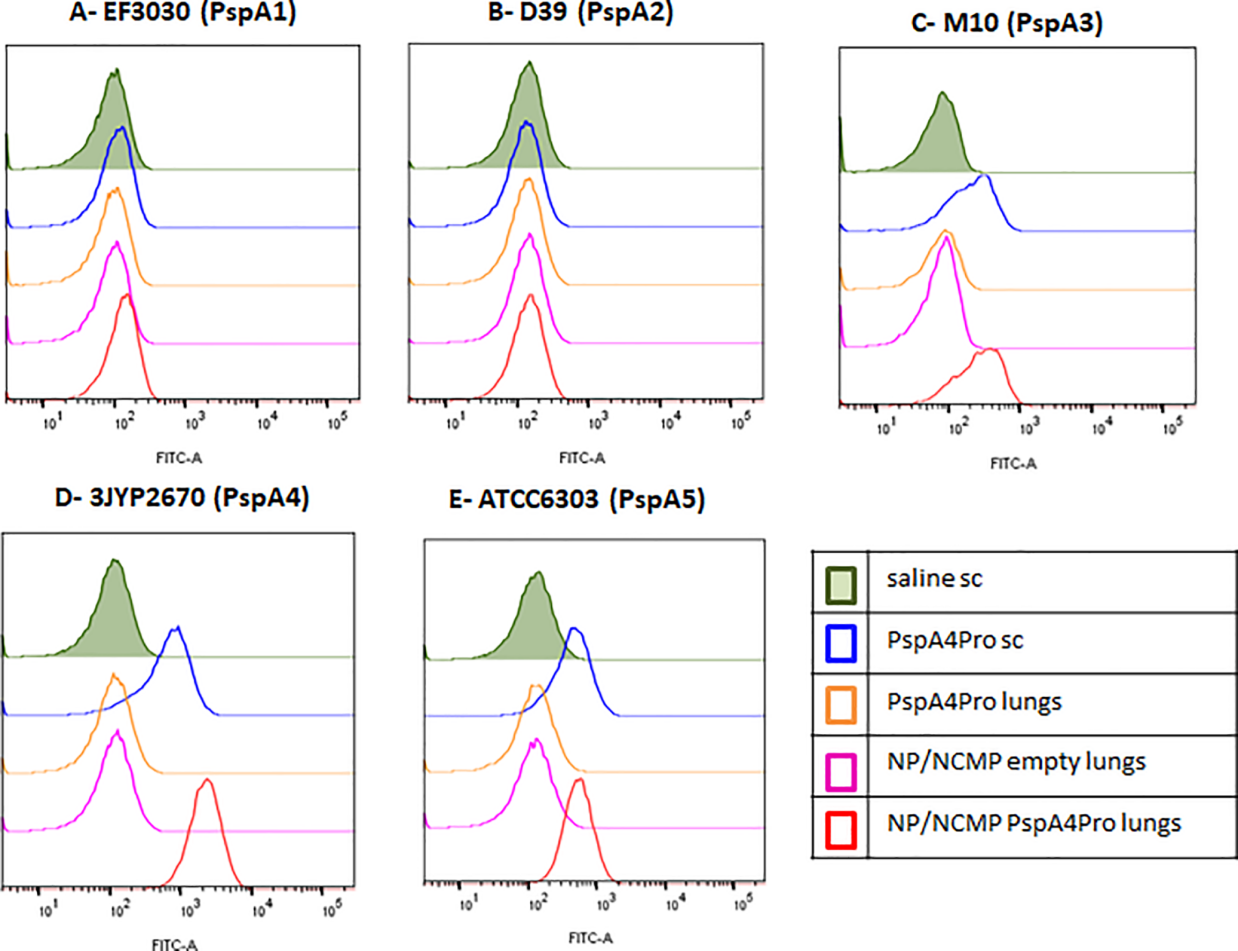
Binding of serum IgG to intact pneumococci. Sera from mice immunized with two doses of the indicated formulations were tested for the ability to bind to pneumococcal strains expressing PspA from clades 1 (A), 2 (B), 3 (C), 4 (D) and 5 (E). Results are shown as fluorescence intensity histograms and are representative of two experiments using sera from independent immunizations.

Binding of serum IgG antibodies to pneumococci from different serotypes and expressing PspA from clades 1 to 5 was analyzed by flow cytometry. Antibodies elicited by immunization with PspA4Pro sc and NP/NCMP PspA4Pro did not bind to strains expressing PspA from family 1, EF3030 (serotype 19F, PspA1) or D39 (serotype 2, PspA2). On the other hand, binding to strains expressing PspA from family 2 was observed, with binding to M10 (serotype 11A, PspA3) and high binding to 3JYP2670 (serotype 3, PspA4) and ATCC6303 (serotype 3, PspA5) (Fig 3, S1 Table).

### Pneumococcal lethal challenge with strain ATCC6303 (serotype 3, PspA5)

**Fig 4:**
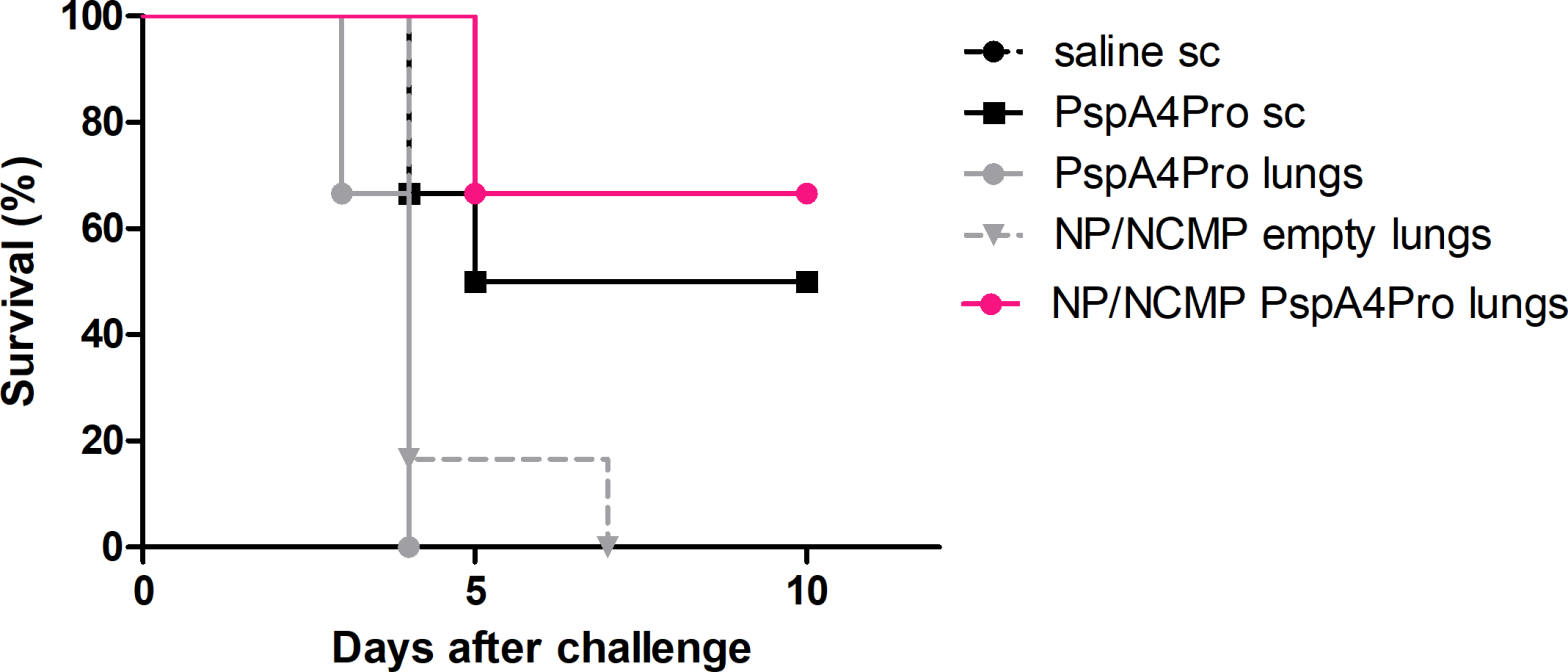
Survival curve of mice challenged with ATCC6303. Mice were immunized with the indicated formulations and challenged with pneumococcal strain ATCC6303. Survival was monitored for 10 days. * indicates statistically significant difference with saline (Log-rank (Mantel-Cox) test).

Efficacy of lung immunization with NP/NCMP PspA4Pro was then tested against a lethal challenge with pneumococcal strain ATCC6303 delivered to the lungs 21 days after the second dose. Partial protection was observed for mice immunized with PspA4Pro sc (50%–3 out of 6, *p* = 0.09) and NP/NCMP PspA4Pro (67%–4 out of 6, *p* = 0.03) (Table 1, Fig 4).

**Table 1:** Survival after lethal challenge with ATCC6303.

	alive/total	% survival	*P* *
**1- saline sc**	0/6	0	-
**2- PspA4Pro sc**	3/6	50	0.09
**3- PspA4Pro lungs**	0/6	0	-
**4- NP/NCMP empty lungs**	0/6	0	-
**5- NP/NCMP PspA4Pro lungs**	4/6	67	0.03

* Fisher exact test

**Fig 5:**
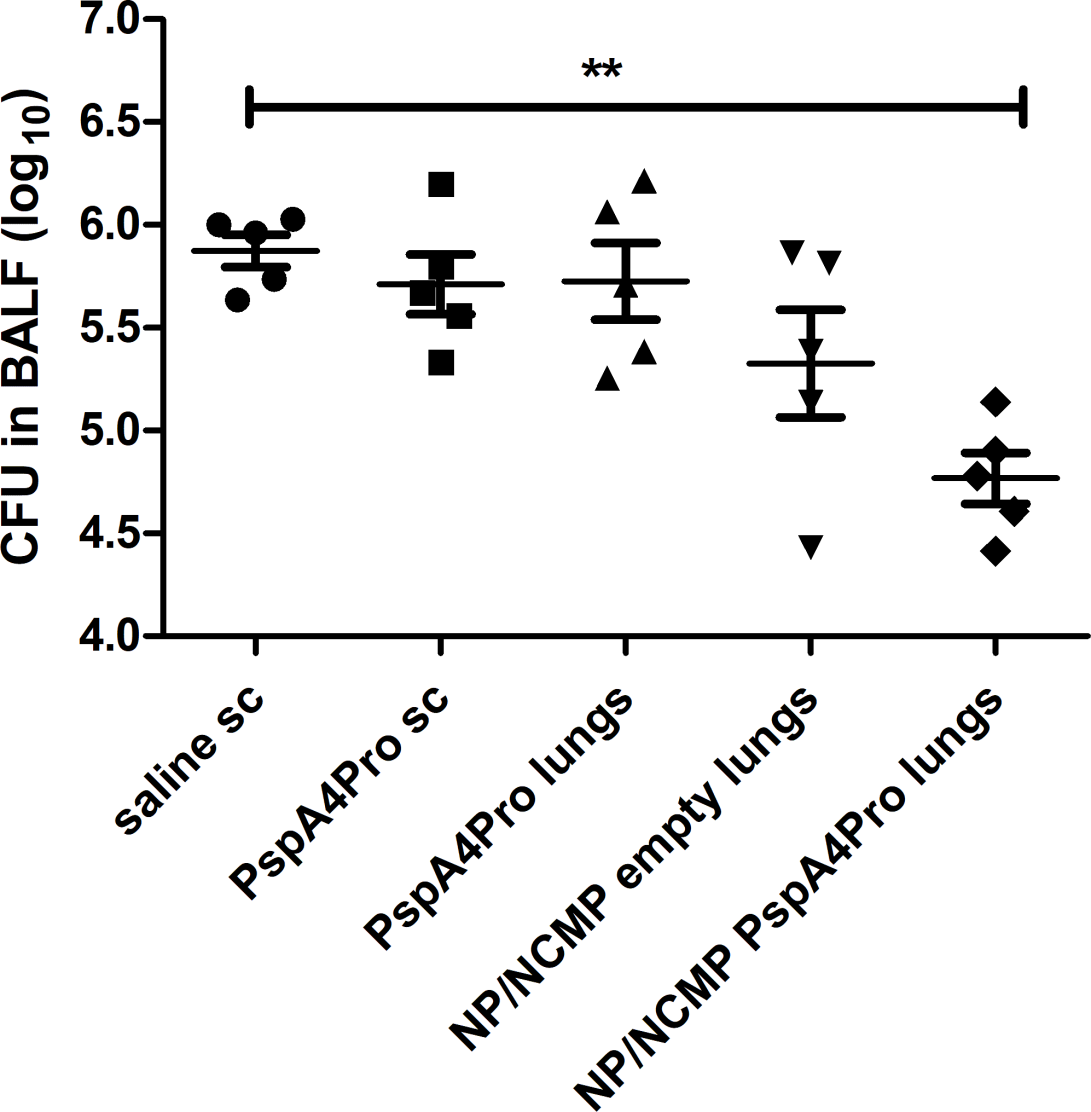
Pneumococcal load in BALF collected 12 hours after lethal challenge with ATCC6303. Mice were immunized with 2 doses of the indicated formulations and challenged with pneumococcal strain ATCC6303. Pneumococcal load in BALF was determined 12 hours after challenge. * indicates statistically significant difference with saline (One-way ANOVA, Tukey’s Multicomparison Test). Symbols represent each individual. Means±standard errors are shown. Representative of two independent experiments.

Bacterial load in BALF collected 12 hours after challenge was also analyzed. Only animals immunized with NP/NCMP PspA4Pro had significant lower pneumococcal load compared with saline. Approximately 1 log reduction on CFU was observed (Fig 5). Thus, although overall survival after 10 days was similar in animals immunized with PspA4Pro sc and NP/NCMP PspA4Pro, the group inoculated with NP/NCMP PspA4Pro showed an earlier response in controlling the infection at 12 hours after challenge. Some reduction in the group inoculated with NP/NCMP empty was also observed at this early phase, indicating the influence of non-specific responses at this time of infection.

**Fig 6:**
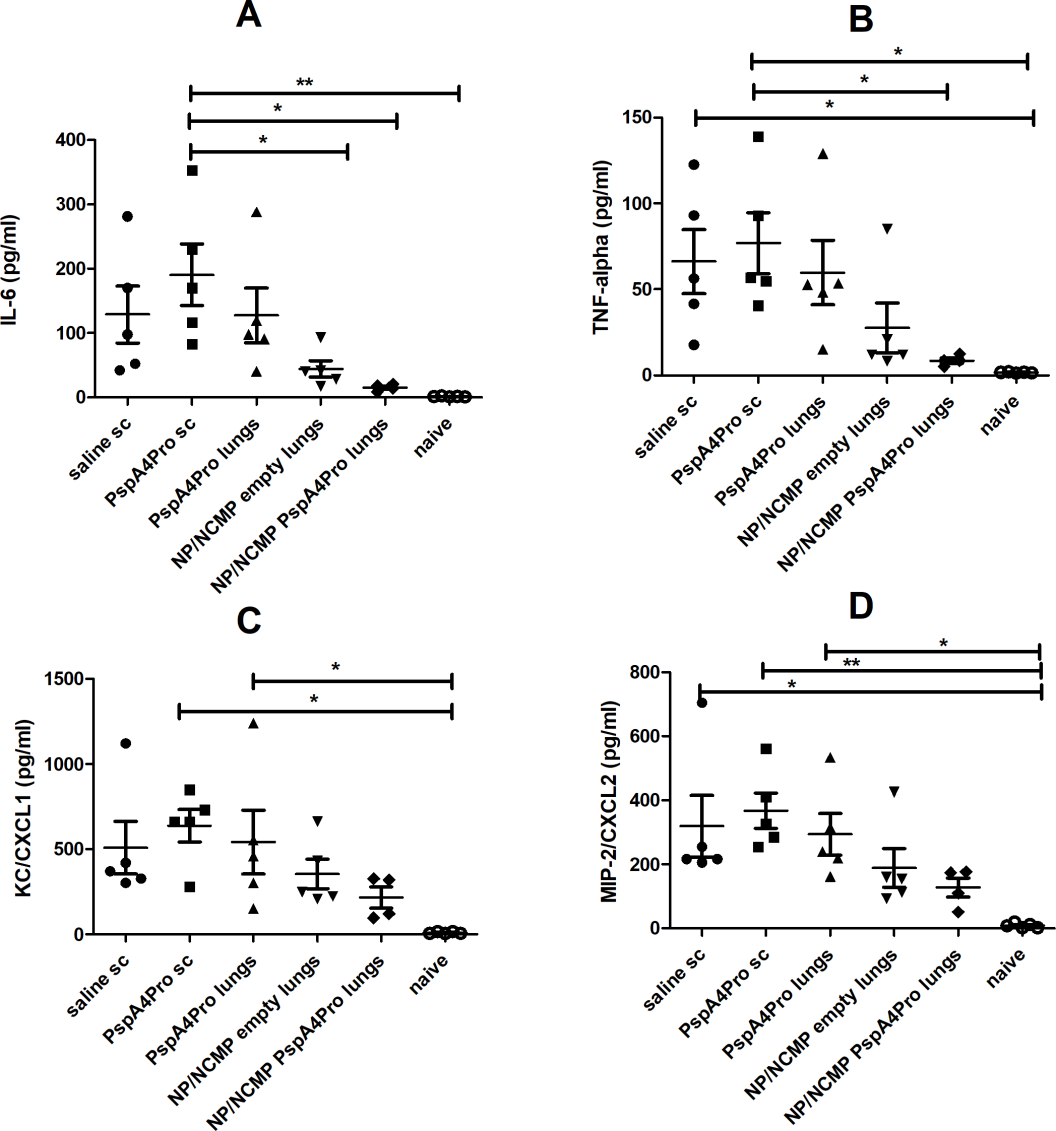
Cytokine/chemokine levels in BALF collected 12 hours after lethal challenge with ATCC6303. Mice were immunized with 2 doses of the indicated formulations and challenged with pneumococcal strain ATCC6303. Levels of IL-6 (A), TNF-α (B), KC/CXCL1 (C) and MIP-2/CXCL2 (D) were determined in BALF collected 12 hours after challenge by Luminex. Naive mice were not immunized nor challenged. * indicates statistically significant difference (One-way ANOVA, Tukey’s Multicomparison Test). Symbols represent each individual. Means±standard errors are shown. Representative of two independent experiments.

Cytokine/chemokine content in BALF was also analyzed. Levels of IFN-γ, IL-1-β, IL-5, IL-6, IL-10, IL-17A, KC/CXCL1, MCP-1/CCL-2, MIP-2/CXCL2 and TNF-α were measured. We observed differences between groups only for the proinflammatory cytokines IL-6 (Fig 6A) and TNF-α (Fig 6B) and for the neutrophil chemoattractants KC/CXCL1 (Fig 6C) and MIP-2/CXCL2 (Fig 6D). Overall, higher levels of these cytokines and chemokines were observed for PspA4Pro sc compared to naive mice (not immunized nor challenged). These results are in accordance with protection elicited by immunization with PspA4Pro sc and NP/NCMP PspA4Pro progressing differently, with higher inflammatory responses and pneumococcal load in the PspA4Pro sc group after 12 hours.

**Fig 7:**
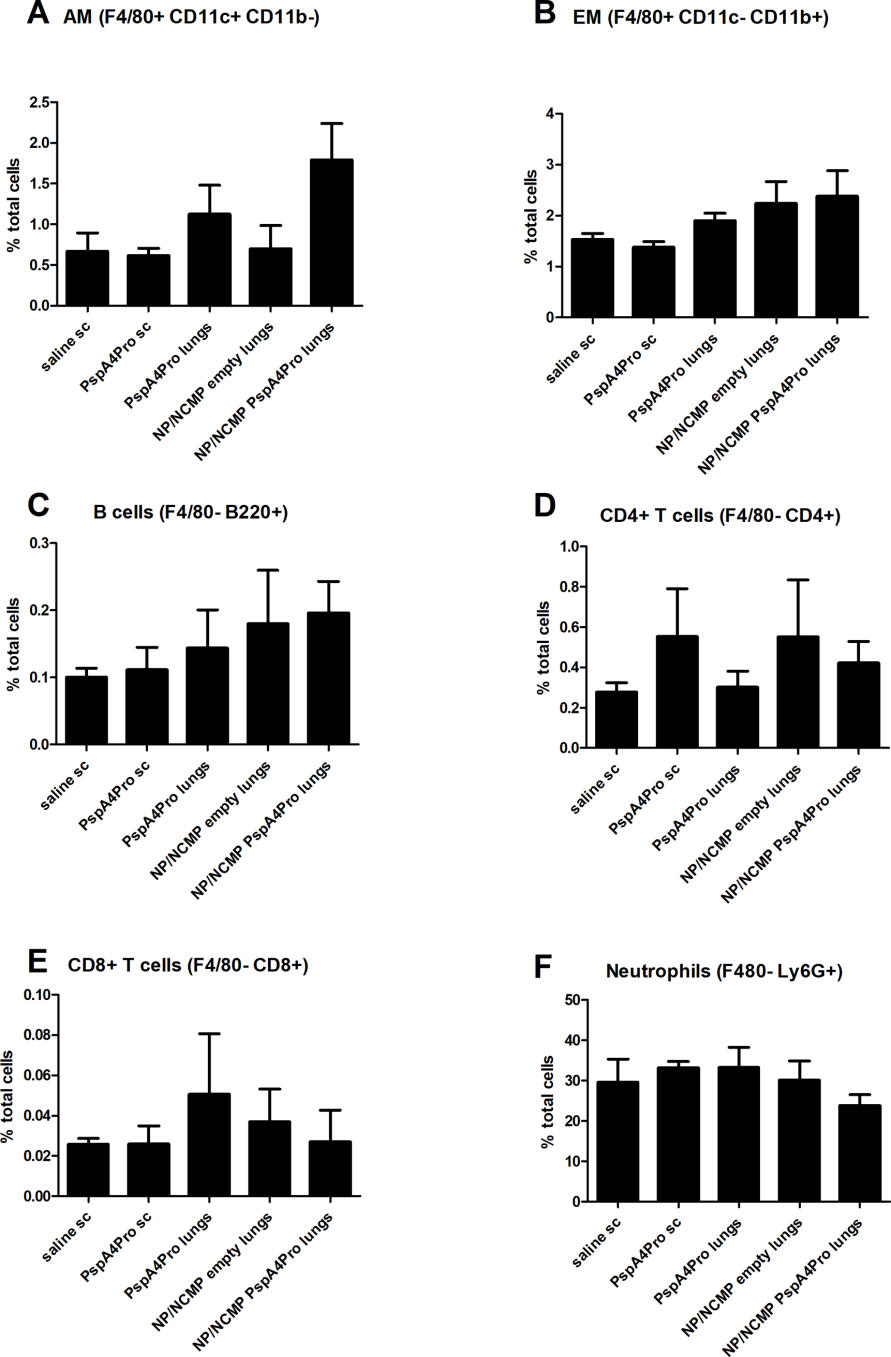
Immunophenotyping of cells recovered in BALF collected 12 hours after lethal challenge with ATCC6303. Mice were immunized with 2 doses of the indicated formulations and challenged with pneumococcal strain ATCC6303. BALF was collected 12 hours after challenge and recovered cells were immunophenotyped by flow cytometry. Percentages of alveolar macrophages (AM—F4/80+ CD11c+ CD11b-) (A), exudate macrophages (EM—F4/80+ CD11c- CD11b+) (B), B cells (F4/80- B220+) (C), CD4+ T cells (F4/80- CD4+) (D), CD8+ T cells (F4/80- CD8+) (E) and neutrophils (F4/80- Ly6G+) (F) are shown. Means and standard errors of each group are shown.

Immunophenotyping of the cells infiltrated in BALF 12 hours after challenge was also performed (Fig 7). Percentage of alveolar macrophages (AM—F4/80^+^ CD11c^+^ CD11b^-^), exudate macrophages (EM—F4/80^+^ CD11c^-^ CD11b^+^), B cells (F4/80^-^ B220^+^), CD4^+^ T cells (F4/80^-^ CD4^+^), CD8^+^ T cells (F4/80^-^ CD8^+^) and neutrophils (F4/80^-^ Ly6G^+^) were analyzed, but no statistically significant differences were observed. In all groups, the majority of the cells were neutrophils that infiltrated after the challenge. A trend toward higher percentage of AMs, EMs and B cells and lower percentage of neutrophils was observed in animals immunized with NP/NCMP PspA4Pro.

### Pneumococcal non-lethal challenge with strain EF3030 (serotype 19F, PspA1)

**Fig 8:**
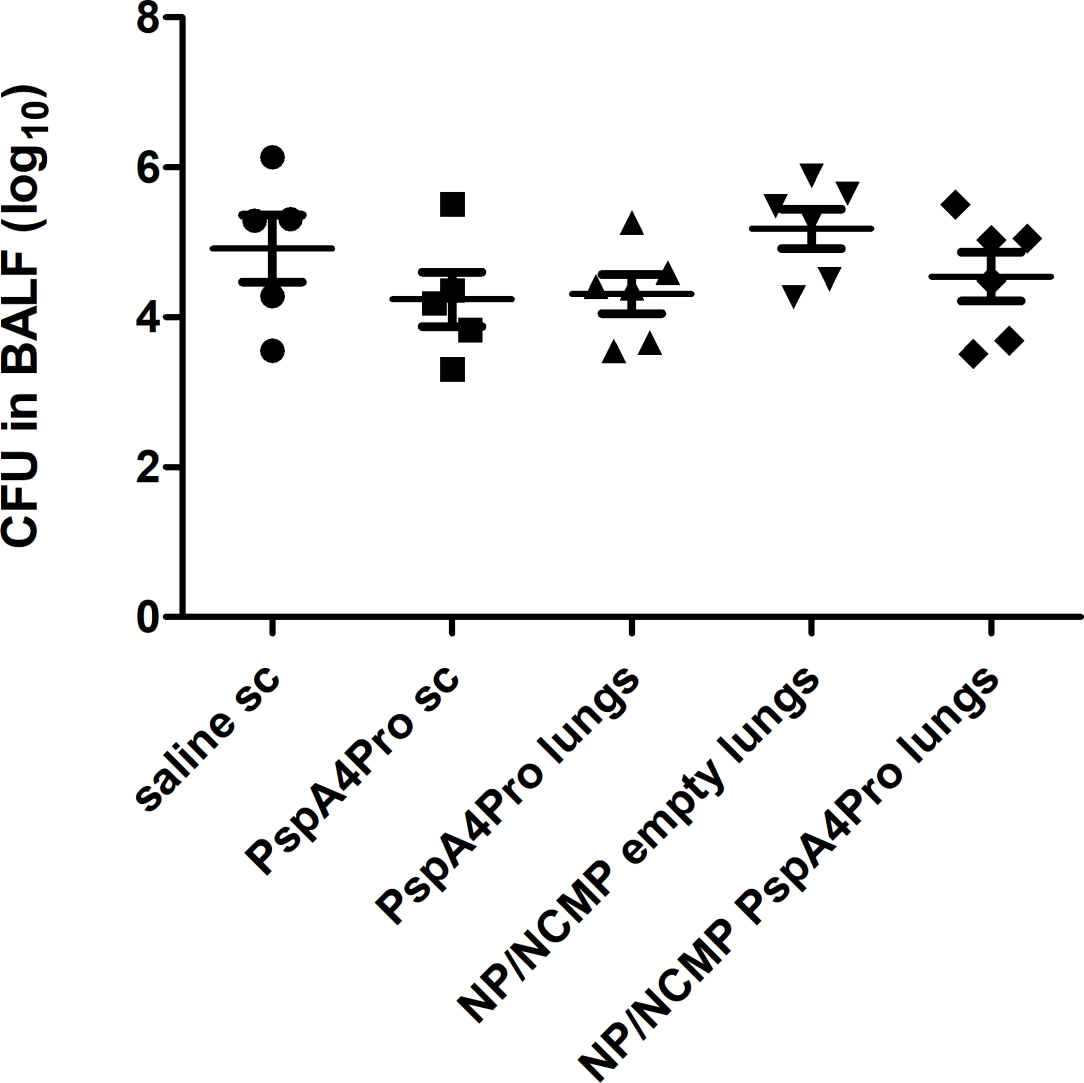
Pneumococcal load in BALF collected 24 hours after non-lethal challenge with EF3030. Mice were immunized with 2 doses of the indicated formulations and challenged with pneumococcal strain EF3030. Pneumococcal load in BALF was determined 24 hours after challenge. Symbols represent each individual. Means±standard errors are shown.

Efficacy of immunization with NP/NCMP PspA4Pro was then tested against lung challenge with pneumococcal strain EF3030, which does not lead to the death of BALB/c mice. Bacterial load in BALF was assessed 24 hours after challenge and no differences were observed between groups (Fig 8).

## Discussion

We have evaluated a dry powder formulation composed of NCMPs containing PGA-co-PDL NPs adsorbed with PspA4Pro for mucosal vaccination targeting the lungs against pneumococcal infection. A dry-powder vaccine for humans could be transported without cold-chain requirements and could then be delivered to the lungs using an inhaler as powder or using a nebulizer after resuspension in liquid vehicle.

Recently, the importance of regionally compartmentalized resident memory CD4^+^ T cells was described in the naturally acquired protection against pneumococcal pneumonia, with superior local tissue protection compared to systemic or central memory responses [23]. Furthermore, previous sub-lethal lung infection of mice with pneumococci was shown to provide better protection against pneumococcal pneumonia by heterologous strains when compared to colonization, emphasizing the importance of local lung immune responses in the protection against pneumonia [24].

Pulmonary delivery of the 23-valent pneumococcal polysaccharide vaccine with a nebulizer has been previously tested in chronic obstructive pulmonary disease patients and healthy adults and lower responses were observed compared to intramuscular injection [25, 26]. Another study using a similar immunization strategy reported no induction of IgG or IgA antibodies in serum or BALF [27]. The 23-valent vaccine however has non-conjugated polysaccharides and induces T-independent responses that tend to be weaker than immune responses to protein antigens.

For experimental mucosal delivery of NP/NCMP PspA4Pro targeting the lungs of mice, the resuspension of the formulation was inoculated through instillation with a large volume. Nasal administration with small volumes (~10 μl) is routinely used in our laboratory for immunization targeting nasal tissue in mice, whereas larger volumes (40–50 μl) ensure delivery to the lungs. Although we cannot rule out the induction of part of the immune responses in the nasal tissue, the largest amount of antigen is delivered to the lungs using this procedure. Furthermore, the use of nebulizers in humans for lung immunization would also deliver the largest amount of the vaccine to the lungs, but some of the inoculum would also be delivered to the nasopharynx and/or oropharynx, similarly to our experimental procedure in mice. High anti-PspA4Pro IgG titers were detected in serum after two doses of NP/NCMP PspA4Pro targeting the lungs. Titers were higher than sc inoculation of purified PspA4Pro and, importantly, instillation of purified PspA4Pro induced a negligible antibody response, indicating that formulation of the protein in NPs is essential for the immune response. Anti-PspA4Pro IgG, but not IgA, was detected in BALF of mice immunized with NP/NCMP PspA4Pro.

Analysis of the overall survival after lethal pneumococcal challenge with ATCC6303 (serotype 3, PspA5) showed the induction of partial protection by NP/NCMP PspA4Pro and by subcutaneous inoculation of the purified protein. Furthermore, 12 hours after pneumococcal challenge, lower bacterial loads were detected in BALF of mice immunized with NP/NCMP PspA4Pro. At this time point, levels of IL-6, TNF-α, KC/CXCL1 and MIP-2/CXCL2 in BALF were higher in animals immunized with PspA4Pro sc compared to naive mice. These results indicate that although these immunization strategies induce similar final survival levels, lung immunization with protein-adsorbed NP/NCMPs induces an earlier control of the infection. It is possible that the higher anti-PspA4Pro IgG titers seen in BALF after immunization with NP/NCMP PspA4Pro into the lungs compared to PspA4Pro sc contribute to the earlier control of infection in that group. These results are in accordance with previous data from our group showing that protection against lethal pneumococcal challenge elicited by nasal immunization with a mixture of recombinant PspA and whole-cell pertussis vaccine correlated with early and controlled inflammatory responses in the lungs [28]. Analysis performed at several time points after challenge showed that protected animals had a controlled cellular infiltration, with neutrophil infiltration between 6 and 24 hours after challenge, peaking at 12 hours. Furthermore, there was controlled secretion of inflammatory cytokines in the lungs of immunized animals, with lower levels of IL-6 and TNF-α in BALF starting at 12 hours post challenge compared to control groups [28]. Studies using naive mice have also shown elevated levels of IL-6, IL-1β, MCP-1, MIP-1, MIP-2 and KC in lung tissue starting at 24 hours post challenge in animals that do not control pneumococcal infection [29]. We did not observe any statistically significant difference between experimental groups in the immune cell subtypes infiltrated in BALF and this can be attributed to the time point used to phenotype cell subsets. It is interesting to note that the group immunized with NP/NCMP empty also showed lower bacterial load and reduced levels of proinflammatory cytokines and neutrophil chemoattractant chemokines at early stages of infection, indicating a role for non-specific responses. This early control of infection is not maintained at longer times after challenge though, since overall survival was not increased in this group. Polymeric NPs were previously shown to have adjuvant properties, which might explain the induction of non-specific innate responses by NP/NCMP empty [7].

Neither immunization with PspA4Pro sc nor NP/NCMP PspA4Pro led to reduced bacterial burden in BALF 24 hours after challenge with strain EF3030 (serotype 19F, PspA1), which was used in a non-lethal mouse model of pneumococcal infection. Immunization with NP/NCMP PspA4Pro thus induced protection against a strain expressing PspA from the homologous family 2 (ATCC6303, PspA5), but not against a strain expressing PspA from the heterologous family 1 (EF3030, PspA1). These results are in accordance with the binding of antibodies to strains expressing PspA from family 2 (PspA3, PspA4 and PspA5), but not to strains expressing PspA from family 1 (PspA1 and PspA2). The inclusion of PspAs from both families has indeed been proposed for a vaccine formulation with broad coverage [11]. Our group has shown that PspA4 is able to induce antibodies with broad reactivity [12], but this previous work was done using parenteral immunization with alum as adjuvant.

Since only protection against pneumococcal challenge with a strain expressing PspA from the homologous family was observed, further improvements in the formulation are needed. Besides the inclusion of a PspA from family 1, NPs composed of polymers other than PGA-co-PDL and/or the inclusion of adjuvants to the formulations may help in improving the immune response. In fact, the use of a TLR2-ligand adjuvant in a formulation delivered to the lungs elicited protective responses in mice against an aerosol challenge with *Mycobacterium tuberculosis* [30]. Delivery to the lungs of NPs conjugated with the tuberculosis antigen Ag85B with the adjuvant CpG elicited Th1 and Th17 responses that also led to enhanced protection against aerosol challenge with *M*. *tuberculosis* [31].

Vaccination targeting the lungs is thus a promising alternative to induce local immune responses against respiratory pathogens, including *M*. *tuberculosis*, influenza virus and *S*. *pneumoniae*. Furthermore, it is important to emphasize that lung immunization in humans is currently feasible due to the availability of several disposable devices. A large trial comparing measles vaccine by means of either aerosol inhalation with a nebulizer or subcutaneous injection has already been performed in India, as part of the Measles Aerosol Vaccine Project coordinated by WHO [32], showing that mass immunization programs can be performed using lung vaccination.

## Supporting information

S1 Fig Distribution of dye after nasal instillation targeting the lungs. Lungs of a mouse after nasal instillation of 50 μl of 0.05% Evans Blue under anesthesia (B). Lungs of a control mouse are also shown (A).
(PDF)

S1 Table Binding of IgG to intact pneumococci—Median Fluorescence Intensity (MFI). (PDF)
