# Supplementary material for: Mucosal immunization with PspA (Pneumococcal surface protein A)-adsorbed nanoparticles targeting the lungs for protection against pneumococcal infection
Source: PLoS One. 2018 Jan 23;13(1):e0191692. doi: 10.1371/journal.pone.0191692 (PMC5779684; doi:10.1371/journal.pone.0191692)
Supplement: S1 Fig — Lungs of a mouse after nasal instillation of 50 μl of 0.05% Evans Blue under anesthesia (B). Lungs of a control mouse are also shown (A). (PDF) [file pone.0191692.s001.pdf]

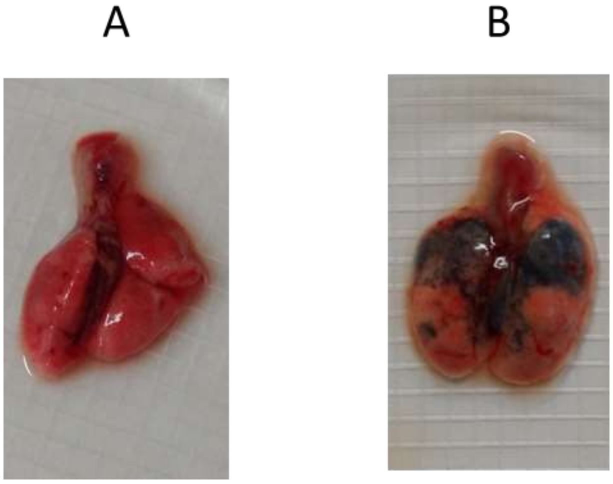

S1 Fig. Distribution of dye after nasal instillation targeting the lungs. Lungs of a mouse after nasal instillation of 50 µl of 0.05% Evans Blue under anesthesia (B). Lungs of a control mouse are also shown (A).
