# Supplementary material for: Mucosal immunization with PspA (Pneumococcal surface protein A)-adsorbed nanoparticles targeting the lungs for protection against pneumococcal infection
Source: PLoS One. 2018 Jan 23;13(1):e0191692. doi: 10.1371/journal.pone.0191692 (PMC5779684; doi:10.1371/journal.pone.0191692)
Supplement: S1 Table — (PDF) [file pone.0191692.s002.pdf]

S1 Table. Binding of IgG to intact pneumococci - Median Fluorescence Intensity (MFI)

	EF3030	D39	M10	3JYP2670	ATCC6303
**saline sc**	86	127	71	104	119
**PspA4Pro sc**	103	122	201	657	432
**PspA4Pro lungs**	88	126	76	108	120
**NP/NCMP empty lungs**	90	130	75	109	116
**NP/NCMP PspA4Pro lungs**	127	135	262	2121	513
